# Quantum ferroelectricity in charge-transfer complex crystals

**DOI:** 10.1038/ncomms8469

**Published:** 2015-06-16

**Authors:** Sachio Horiuchi, Kensuke Kobayashi, Reiji Kumai, Nao Minami, Fumitaka Kagawa, Yoshinori Tokura

**Affiliations:** 1 National Institute of Advanced Industrial Science and Technology (AIST), Tsukuba 305-8562, Japan; 2 CREST, Japan Science and Technology Agency (JST), Tokyo 102-0076, Japan; 3 Condensed Matter Research Center (CMRC) and Photon Factory, Institute of Materials Structure Science, High Energy Accelerator Research Organization (KEK), Tsukuba 305-0801, Japan; 4 Department of Applied Physics, The University of Tokyo, Tokyo 113-8656, Japan; 5 RIKEN Center for Emergent Matter Science (CEMS), Wako 351-0198, Japan

## Abstract

Quantum phase transition achieved by fine tuning the continuous phase transition down to zero kelvin is a challenge for solid state science. Critical phenomena distinct from the effects of thermal fluctuations can materialize when the electronic, structural or magnetic long-range order is perturbed by quantum fluctuations between degenerate ground states. Here we have developed chemically pure tetrahalo-*p*-benzoquinones of *n* iodine and 4–*n* bromine substituents (QBr_4–*n*_I_*n*_, *n*=0–4) to search for ferroelectric charge-transfer complexes with tetrathiafulvalene (TTF). Among them, TTF–QBr_2_I_2_ exhibits a ferroelectric neutral–ionic phase transition, which is continuously controlled over a wide temperature range from near-zero kelvin to room temperature under hydrostatic pressure. Quantum critical behaviour is accompanied by a much larger permittivity than those of other neutral–ionic transition compounds, such as well-known ferroelectric complex of TTF–QCl_4_ and quantum antiferroelectric of dimethyl–TTF–QBr_4_. By contrast, TTF–QBr_3_I complex, another member of this compound family, shows complete suppression of the ferroelectric spin-Peierls-type phase transition.

## Abstract (web summary)

Quantum critical behaviour emerges when quantum fluctuations perturb the balance between electronic states of a material having the same energy, and can lead to novel phenomena. Here, the authors discover quantum criticality in the ferroelectric behaviour of organic molecular solids.

## Graphical abstract

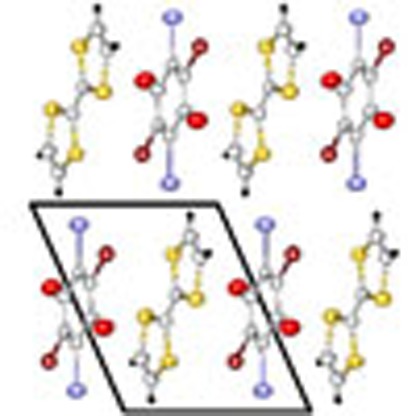

Lattice instability coupled with charge and/or spin degrees of freedom has become an interesting area of research in solid state science as the electric, magnetic and optical properties exhibit high susceptibility to external stimuli. One intriguing characteristic is ferroelectricity susceptible to changes in temperature, pressure and external electric field. Typical examples among π-conjugated molecular solids are charge-transfer (CT) complexes^1^,^2^,^3^,^4^,^5^,^6^,^7^,^8^ that have one-dimensional stacks of alternating π-electron donor (D) and acceptor (A) molecules, as in a series of complexes of *p*-benzoquinones with tetrathiafulvalene (TTF) or its 4,4′-dimethyl-substituted version (DMTTF). The bistable polarity along the molecular stack can be constructed by a pairwise molecular displacement (dimerization denoted by underlines such as DA DA.. or AD AD …).

There are at least two different classes of ferroelectrics in TTF complexes. The emergence of ferroelectricity in TTF–QCl_4_ (ref. 9; QCl_4_ or CA=*p*-chloranil) and antiferroelectricity in DMTTF–QCl_4_ (ref. 10) accompanies a phase transition from a neutral to ionic state under a temperature or pressure change. The charge degree of freedom is thus closely relevant to the mechanism of the neutral–ionic phase transition (NIT). The crystal volume is an effective parameter to characterize the NIT, because it can change the energy balance between the electrostatic Madelung gain and the cost of ionization during increasing the degree of CT *ρ* (ionicity defined by D^+*ρ*^A^–*ρ*^)^9^. The recent discovery of large spontaneous polarization on TTF–QCl_4_ has highlighted a new mechanism of ferroelectric induction, ‘electronic ferroelectricity', that is governed by the electronic overlap integral over the intermolecular space rather than the static molecular charge^3^,^11^,^12^,^13^. Note that the recent studies on organic CT compounds also demonstrated gigantic permittivity from the charge-order mechanism as the similar class of electronic ferroelectricity^14^.

The contrastive ferroelectric, the bromine-substituted analogue, TTF–QBr_4_ (QBr_4_=*p*-bromanil)^15^ exhibits a similar DA dimerization but preserves the monovalent ionic state during the ferroelectric transition. The antiferromagnetically coupled *S*=1/2 spins residing on D^+^ and A^–^ radical ions turn to spin-singlet D^+^A^–^ pairs on cooling, changing the chains from paramagnetic to nonmangetic. This ferroelectricity, thus, has a magnetic origin corresponding to the spin-Peierls (SP)-type phase transition that is manifested by the magnetoelectric phenomena such as magnetic control of electric polarization^16^.

For both NIT and SP systems, one challenging issue is the quantum phase transition^17^ achieved by fine tuning the continuous phase transition down to zero kelvin. New states of matter may materialize when the electronic, structural and/or magnetic long-range order is perturbed by quantum fluctuations such as the ‘zero-point motion' between the degenerate ground states as opposed to thermal fluctuations. The ‘quantum ferroelectric' and ‘quantum paraelectric' states represent those in proximity to the zero-kelvin transition point, that is, the quantum critical point (QCP)^18^,^19^,^20^,^21^,^22^. For instance, in the typical quantum paraelectric SrTiO_3_, the temperature-dependent dielectric permittivity exhibits a saturation behaviour at a gigantic value (∼20,000) as the manifestation of the proximity to the QCP, and quantum ferroelectricity can be induced with subtle structural perturbations such as oxygen isotope exchange^21^. Although a quantum critical behaviour has been found in the NIT system by pressurizing DMTTF–QBr_4_ crystals, the inter-stack antiferroelectric coupling, which leads to the antiferroelectrically ordered state, is responsible for the much smaller permittivity (∼180) at the QCP.

To search for the QCP accompanied by quantum ferroelectricity, tetrahalo-*p*-benzoquinones of variable molecular volume are ideal for inducing structural changes with least perturbations to the electron affinities. In fact, the NIT has been demonstrated through optical spectra of two neutral TTF–QI_4_ complexes of different stoichiometries (1:1 and 2:1) under a modest pressure of ∼2–3 GPa^23^,^24^,^25^. In this study, we prepared tetrahalo-*p*-benzoquinones with *n* iodine and 4–*n* bromine substituents (QBr_4–*n*_I_*n*_, *n*=0–4; Fig. 1a) to examine the stepwise change in the lattice volume and to modify the NIT of TTF–QI_4_ and the ferroelectric SP of TTF–QBr_4_. In this family, we have realized the ideal NIT type quantum ferroelectricity (TTF–QBr_2_I_2_) by tuning the applied hydrostatic pressure

## Results

### Materials preparation

Considering that even small chemical inhomogeneity will mask the genuine properties around the QCP, we pursued QBr_4–*n*_I_*n*_ molecules with sufficient chemical purity for fine and systematic chemical tuning. However, previous synthetic protocols did not include the regioselective iodination step. This work established an effective purification method using gel permeation chromatography and regioselective iodination procedures, the details of which are described in the Supplementary Fig. 1. Gel permeation chromatography isolated 2-iodo-3,5,6-tribromo-*p*-benzoquinone (QBr_3_I) and 2-bromo-3,5,6-triiodo-*p*-benzoquinone (QBrI_3_) as well as pure QI_4_, but the dibromodiiodo-*p*-benzoquinones could not be separated into three isomers. The symmetrically diiodo-substituted quinone (QBr_2_I_2_) was synthesized through the new regioselective iodination process using symmetric dibromodimethoxybenzene.

### CT complex formation

**Figure 1:**
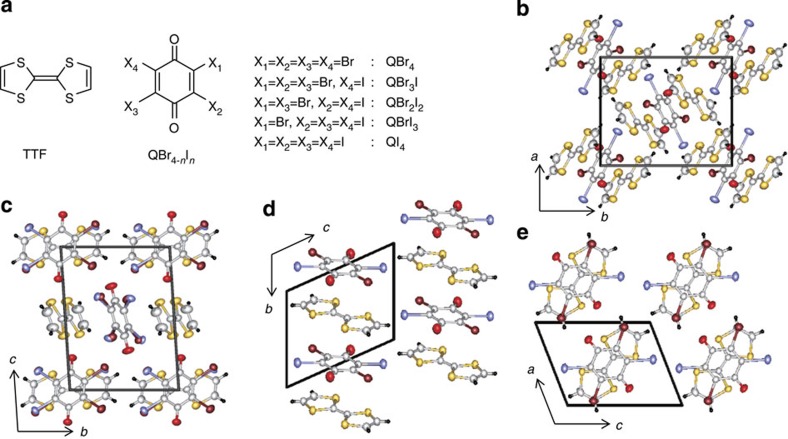
Chemical and crystal structures of tetrathiafulvalene (TTF) complexes of tetrahalo-*p*-benzoquinones (QBr_4–*n*_I_*n*_). (**a**) Chemical form of QBr_4–*n*_I_*n*_. (**b-e**) Molecular packings of the TTF–QBr_4–*n*_I_*n*_ complexes. (**b**) Neutral (TTF)_2_(QBr_2_I_2_) projected along the *c* direction. (**c**) Ionic 1:1 TTF–QBr_3_I projected along the *a* direction. (**d**,**e**) Neutral 1:1 TTF–QBr_2_I_2_ projected along the crystallographic *a* and *b* directions. For clarity, the halogen positions of the QBr_2_I_2_ molecules are shown only for preferable occupations.

Without seeding specific crystal forms, the mixed acetonitrile solution of each QBr_4–*n*_I_*n*_ and TTF afforded neutral CT complexes of a 2:1 stoichiometry as the main products. The crystal structures of (TTF)_2_(QBr_4–*n*_I_*n*_) are isomorphous and belong to the monoclinic system with a centric space group of *P*2_1_/*n*. The molecules assemble into a DAD trimer with inversion symmetry, as shown in Fig. 1b. Previous spectroscopic studies of the pressure-induced NIT of (TTF)_2_(QI_4_) suggested an inhomogeneous charge distribution in the ionic phase at high pressures beyond 3.1 GPa^26^. High-pressure structural studies, albeit beyond the focus of this study, would be interesting to confirm the asymmetric charge distribution suggested as a polar D^+^A^–^D^0^ and to examine the possible emergence of electronic ferroelectricity.

The 1:1 TTF complexes of QBr_4–*n*_I_*n*_ with an infinitely alternating DA sequence are usually obtained as minor products. They crystallized as either fully ionic or neutral CT complex depending on the degrees of bromine substitution between the neutral TTF–QI_4_ and ionic TTF–QBr_4_. The overall CT complexes of TTF are listed in terms of the isomorphous crystal forms in Supplementary Table 1.

### Ionic 1:1 TTF complexes

**Figure 2:**
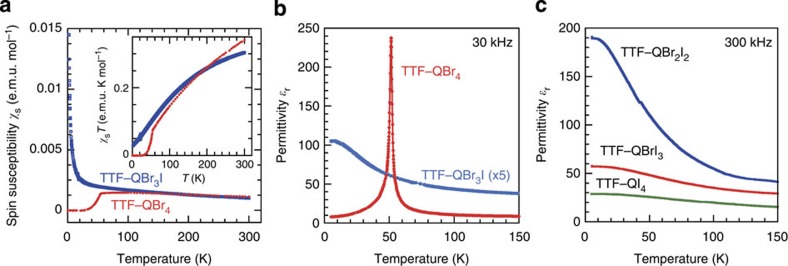
Temperature dependence of dielectric and magnetic properties of 1:1 TTF–QBr_4–*n*_I_*n*_ complexes. (**a**) The spin susceptibility *χ*_s_ for ionic TTF–QBr_4_ and TTF–QBr_3_I crystals. The inset shows the corresponding *χ*_s_*T*–*T* plot. (**b**) The relative permittivity measured with an a.c. electric field applied along the crystallographic *b* axis parallel to the DA stack of ionic TTF–QBr_4_ and TTF–QBr_3_I crystals. (**c**) The relative permittivity measured with an a.c. electric field applied along the DA stack (parallel to the crystallographic *b* axis) for neutral 1:1 TTF–QBr_2_I_2_, TTF–QBrI_3_ and TTF–QI_4_ crystals at ambient pressure.

The 1:1 complex of 2-iodo-3,5,6-tribromo-*p*-benzoquinone (QBr_3_I) crystallizes as an isomorphous form of the fully ionic TTF–QBr_4_ (ref. 15), the SP-type ferroelectric. Crystal structural analysis determined an analogous triclinic lattice with a space group of *P*-1 and two formula units (*Z*=2; Fig. 1c). The D and A molecules, occupying their respective inversion centres, are alternately stacked with a regular separation. The lattice constants of the *a* and *b* axes, which are both parallel to the DA stack, are elongated by 1.2% and 1.1%, respectively, compared with those of the TTF–QBr_4_ crystal. Figure 2a,b depicts the temperature-dependent magnetic susceptibility and dielectric constant of these two isomorphous compounds. The magnetic susceptibility in the high-temperature region indicates the analogous paramagnetism arising from the spin-1/2 residing on each D^+^ and A^−^ radical, but its monotonous increase on cooling in the case of TTF–QBr_3_I represents the absence of the SP transition. In accord with this, the dielectric permittivity has no peak anomaly characteristic of the ferroelectric transition down to the lowest temperature. Nevertheless, the gradual increase in the permittivity with decreasing temperature may reflect the quantum fluctuations of the polar dimerization.

### Neutral 1:1 TTF complexes

The 1:1 TTF complexes of QBr_2_I_2_ and QBrI_3_ with *ρ*∼0.11 at ambient pressure (as estimated from the C=O stretch mode frequency; see Supplementary Discussion) are isomorphous to TTF–QI_4_ (ref. 24) in crystal structure. According to previous studies of high-pressure optical spectra at room temperature, the ionicity of TTF–QI_4_ steeply increases from ∼0.3 to 0.5 and the DA stack starts to dimerize at pressures beyond 1.9 GPa^23^. Because previous studies of TTF–QI_4_ were not accompanied by a full report of the atomic coordinates, we have repeated the structural analysis. The triclinic lattice with a space group of *P*-1 comprises simply one formula unit (*Z*=1). The D and A molecules, occupying their respective inversion centres, are alternately stacked with a regular separation along the crystal *b* direction (Fig. 1d,e). The molecular arrangement is very similar to that of DMTTF–QCl_4_ (ref. 10) in the triclinic cell (*Z*=1) and distinct from that of TTF–QCl_4_ in the monoclinic cell (*Z*=2)^27^.

Bromine substitution of TTF–QI_4_ shortens the D–A distance along the stack, namely, the half of *b* axis lattice constant, and would stabilize the ionic state by chemical pressure effect, or equivalently by increasing the attractive Coulomb interaction between D and A. We have examined the compressibility of the lattice parameters to relate the lattice shrinkage to the effective pressure Δ*p*_eff_ that would contribute to promotion of NIT. Using the volume compressibility of TTF–QBr_2_I_2_ crystal (Δ*V*=–28 Å^3^ at Δ*p*=1 GPa), the volume change from that of TTF–QI_4_ corresponds to the application of Δ*p*_eff_=0.29 GPa for TTF–QBrI_3_ (Δ*V*=–8.2 Å^3^) and 0.60 GPa for TTF–QBr_2_I_2_ (Δ*V*=–16.7 Å^3^). From the *b* axis compressibility (Δ*b*=–0.23 Å at Δ*p*=1 GPa), we obtained similar pressures of 0.29 GPa (Δ*b*=–0.067 Å) and 0.68 GPa (Δ*b*=–0.157 Å), respectively.

### Dielectric properties of neutral 1:1 TTF complexes

Figure 2c depicts the temperature *T* dependence of relative permittivity *ɛ*_r_ measured at 300 kHz along the DA stacking direction of neutral 1:1 compounds. In the displayed temperature range, the permittivity exhibited no frequency dispersion, at least up to 1 MHz. The increase in permittivity with lowering temperature starts to deviate from the Curie–Weiss law for ferroelectrics, *ɛ*_r_=*C*/(*T*–*θ*), where θ is the Weiss temperature and *C* is the Curie constant. The temperature dependence including the saturation behaviour around the lowest temperature has been often fitted with the theoretical (Barrett) formula for quantum paraelectricity^28^:


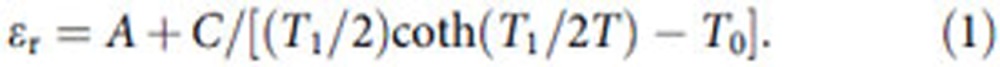


Here *T*_0_ represents the Curie–Weiss temperature in the classical (high *T*) limit, and *T*_1_ is the characteristic crossover temperature dividing the quantum-mechanical and classical regions. The fitting parameters for *T*<140 K are *T*_0_=+3(1) K and *T*_1_=71(1) K; and *C*=6.3(2) × 10^3^ K for TTF–QBr_2_I_2_; and *T*_0_=–62(10) K, *T*_1_=70(4) K and *C*=5.4(7) × 10^3^ K for TTF–QBrI_3_. In the case of TTF–QI_4_, the values are *T*_0_=–165(12) K, *T*_1_=86(2) K and *C*=8.0(7) × 10^3^ K (see the Supplementary Fig. 4 for the fittings). It should be noted that the saturated value of the permittivity as well as *T*_0_ increase with the effective (chemical) pressure noted above, whereas *C* is almost unchanged.

This behaviour is quite analogous to the case of the antiferroelectric NIT of DMTTF–QBr_*n*_Cl_4–*n*_ (ref. 29). Lattice compression under chlorine substitution or hydrostatic pressure increases the low-temperature permittivity until the temperature-induced phase transition appears together with a peak anomaly of the permittivity. The temperature–pressure phase diagram marks the zero-kelvin transition point, that is, the QCP. One may think that decreasing the D–A separation with further bromine substitution beyond TTF–QBr_2_I_2_ would surpass the QCP and realize the temperature-induced ferroelectric NIT at ambient pressure. Unfortunately, this expectation has not yet materialized solely by the chemical modifications because of formation of the ionic CT complexes as noted above. To search for the QCP, we applied the hydrostatic pressure on the TTF–QBr_2_I_2_ crystal.

### TTF–QBr_2_I_2_ under hydrostatic pressure

**Figure 3:**
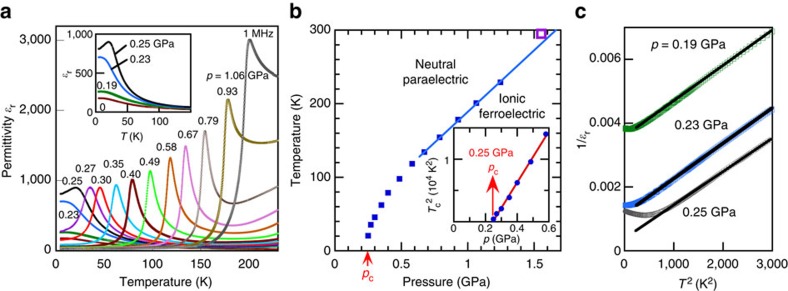
Temperature- and pressure-dependent properties of TTF–QBr_2_I_2_ crystal. (**a**) Temperature dependence of the relative permittivity under various hydrostatic pressures. The applied pressure value, corrected considering its thermal change in the medium for each measurement, is represented by the value at the transition point or lowest temperature when the phase transition is absent. The inset depicts the data in the low-pressure range. (**b**) Temperature–pressure phase diagram; linear extrapolation of the phase boundary at high-temperature region points to 1.64 GPa at room temperature. The inset represents the quantum critical behaviour of ferroelectrics obeying the relation *T*_*c*_∝(*p*–*p*_*c*_)^1/2^ in the low-critical temperature region. The open square represents the pressure at which the room temperature conductivity exhibited a sharp peak caused by the pressure-induced NIT. (**c**) Inverse relative permittivity as the function of the square of the temperature. The solid line represents the quantum critical behaviour *ɛ*_r_^–1^∝*T*^2^.

At ambient and low pressures, the relative permittivity around the lowest temperature (4 K) exhibits saturation behaviour and increases from 200 to 700 with pressure (Fig. 3a). According to the fit with the Barrett formula, *T*_0_ increases to +18.2(6) K at 0.23 GPa with *T*_1_=68.7(7) K and *C*=1.22(2) × 10^5^ K. Although the phase transition is expected near this positive *T*_0_ in the classical picture, it is suppressed by the quantum fluctuations. Beyond the critical pressure of *p*_c_=0.25 GPa, a sharp peak indicative of the phase transition appears and shifts towards a higher temperature with further increasing pressure. The phase transition is accompanied by a small peak anomaly in the temperature dependence of dielectric loss (that is, imaginary part of dielectric constant).

The Barrett formula has a drawback in its theoretical accuracy, although it is a prevailingfitting equation for quantum paraelectricity and convenient for estimating Curie–Weiss temperature in the classical limit as above: it cannot describe the behaviour around the QCP^22^. One of the reasons is that the fluctuation spectrum is assumed to be independent of wave vector and temperature, in contradiction to the observed softening of optical phonon mode in the displacive ferroelectrics. The mode softening has been observed near the ferro- and antiferroelectric transitions also in the family of electronic ferroelectric TTF–CA^3^,^4^,^30^. The fluctuating and softening polar mode in the latter ferroelectric is considered to be the so-called Peierls mode, which is accompanied by the intermolecular electron transfer oscillation coupled with the dimeric molecular displacement. According to the theory of quantum criticality^22^, the inverse dielectric susceptibility should be proportional to the square of temperature near the QCP for the typical displacive ferroelectrics such as SrTiO_3_ and KTaO_3_. In fact, this critical behaviour is clearly seen in the linear *T*^2^-1/ɛ_r_ relation (Fig. 3c) at temperatures below 50 K and pressures near the QCP for the TTF–QBr_2_I_2_ crystal.

Figure 3b shows the pressure–temperature phase diagram obtained from this permittivity measurement. Here the hydrostatic pressure values at the phase transition temperatures are corrected considering the effect of the thermal contraction of the pressure-transmitting oil in the clamp-type high-pressure cell (see Methods). The NIT critical temperature (*T*_c_) tends to increase linearly with pressure in the high-pressure region. Its extrapolation to 1.63 GPa at room temperature (295 K) agrees well with the pressure of 1.55 GPa (marked by an open square in the figure), at which a sharp peak of conductivity (Supplementary Fig. 5) can be related to the pressure-induced NIT; the 0.35 GPa reduction from the pressure required for the NIT of TTF–QI_4_ (∼1.9 GPa)^23^ is roughly explained by the effective pressure of Δ*p*_eff_=0.60 GPa noted above. The large slope (d*T*_c_/d*p*=0.166 K MPa^–1^), which is half that of TTF–QCl_4_ (0.32 K MPa^–1^)^31^, also reflects the pressure-sensitive nature of the NIT.

In contrast, the phase boundary (Fig. 3b) in the low-pressure region exhibits a critical drop, which can be approximated as *T*_c_∝(*p*−*p*_c_)^1/2^. The QCP is well defined at *p*_c_=0.246 GPa through the linear *p*–*T*_c_^2^ relation (see inset in Fig. 3b).

**Figure 4:**
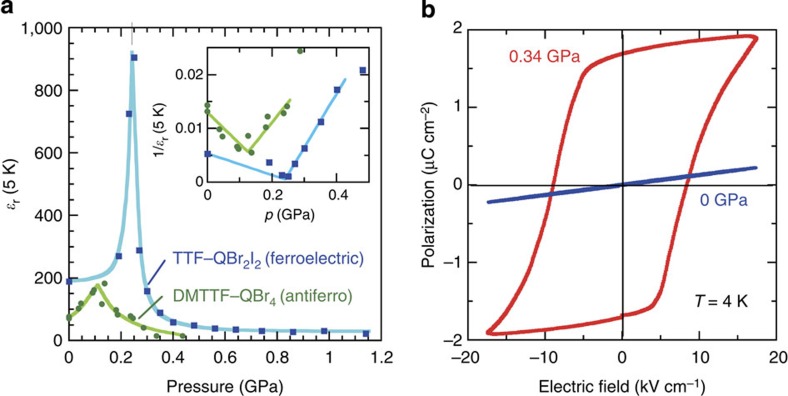
High-pressure properties of TTF–QBr_2_I_2_ crystal at low temperatures. (**a**) Hydrostatic pressure dependence of the relative permittivity at 5 K, *ɛ*_r_ (*T*=5 K) on a TTF–QBr_2_I_2_ crystal (filled squares) in comparison with a quantum (anti)ferroelectric DMTTF–QBr_4_ crystal (filled circles, redrawn based on data in ref. 29). The inset shows the inverse permittivity obeying a simple power law, *ɛ*_r_ (*T*=5 K)∝|*p*–*p*_c_|^–1^, as expected for quantum ferroelectricity. (**b**) Electric polarization (*P*) versus electric field (*E*) hysteresis loops with a triangular a.c. electric field at *T*=4 K and frequency *f*=1 kHz.

The permittivity at the lowest temperature *ɛ*_r_ (*T*=5 K) as a function of pressure reveals a divergent-like sharp maximum at *p*_c_ (Fig. 4a). There are some similarities and dissimilarities in the quantum critical behaviour near the QCP between the present case and the pressure-induced antiferroelectric NIT of DMTTF–QBr_4_ (ref. 29). Despite the very similar crystal structures in the paraelectric state, the inverse-*ɛ*_r_ versus *p* plot (inset to Fig. 4a) indicates the different behaviour between the two crystals around the QCP; in TTF–QBr_2_I_2_, the permittivity increases much more rapidly, obeying a simple power law of *ɛ*_r_ (*T*=5 K)∝|*p*–*p*_c_|^–1^. Because the maximum *ɛ*_r_ (*T*=5 K) of TTF–QBr_2_I_2_ (∼800) is four times as large as that of DMTTF–QBr_4_ (<200), these observations reflect the different nature, that is, ferroelectric versus antiferroelectric, of the pressure-induced ordered phases. This difference manifests itself in structural changes.

Figure 4b presents the electric polarization (*P*) versus electric field (*E*) diagram measured along the DA stacking direction at 4 K. The linear *P*–*E* relation at ambient pressure is characteristic of the paraelectric state. The expected pressure-induced ferroelectric state is clearly demonstrated by the *P*–*E* hysteresis loop at *p*=0.34 GPa. The magnitude of *P*_r_ (1.7 μC cm^–2^ at *p*=0.34 GPa) is >10 times as large as that of the ionic ferroelectric TTF–QBr_4_ (0.15 μC cm^–2^)^16^ but rather close to that of TTF–QCl_4_ (6.3 μC cm^–2^)^12^. This fact suggests that the microscopic origin of ferroelectricity, in particular its electronic nature as mentioned above, is similar in TTF–QCl_4_ and TTF–QBr_2_I_2_.

According to the X-ray diffraction experiments with TTF–QBr_2_I_2_ at 7 K under several different pressures (0.61, 0.67, 1.40, 1.80 and 2.27 GPa), the low-temperature, high-pressure phase is not accompanied by the appearance of any satellite reflections arising from superlattice. Because the triclinic unit cell involves simply one formula unit (*Z*=1), the symmetry-breaking DA dimerization therein confirms its ferroelectric nature. This structural change is in sharp contrast to the antiferroelectric change of unit-cell doubling in the DMTTF complexes^29^. The full structural analysis of the high-pressure phase is under examination and will be reported elsewhere.

## Discussion

QBr_4_ has been successfully substituted with iodine in a stepwise and regioselective manner. The TTF–QBr_2_I_2_ complex has enabled us to continuously control the ferroelectric NIT over a wide temperature range from zero kelvin to room temperature. In past studies, the TTF–QCl_4_ complex was partially replaced with tetraselenafulvalene (TSF), which has a larger molecular volume, to reduce the ferroelectric NIT temperature^32^. Quantum fluctuations are generally fragile against the inhomogeneity of the system, which cannot be removed in terms of the ionization energy in ternary compounds such as TTF_1–*x*_TSF_*x*_–QCl_4_. The binary compound DMTTF–QBr_4_ overcame this problem, and clear quantum critical behaviour of the NIT was exhibited, although the ionic DA lattice is dimerized into an antiferroelectric structure, cancelling out the polarization between neighbouring chains^29^. Renewed interest in the NIT system has also originated from the large electric polarization of TTF–QCl_4_ arising from the intermolecular CT process, namely, ‘electronic ferroelectricity'. The TTF–QBr_2_I_2_ complex is free from inhomogeneities such as the site energy variations and disordered molecular dipoles. TTF–QBr_2_I_2_ exhibits quantum critical behaviours relevant to the ferroelectric order, and hence can be considered as the first clean example of a ‘quantum electronic ferroelectric'.

While the temperature-induced NIT would be expected for a lattice-contracted isomorph of TTF–QBr_2_I_2_, the TTF–QBr_3_I complex forms a different type of crystal structure as another interesting class of quantum ferroelectrics. The monoiodination of the paramagnetic TTF–QBr_4_ salt is accompanied by a complete suppression of the ferroelectric SP transition. The lattice expansion and/or random orientation with dipolar QBr_3_I molecules may be plausible origins of the modified ground state. The ferroelectric SP state of TTF–QBr_4_ is suppressed under a strong magnetic field, exemplifying the ‘organic multiferroelectric'. Further chemical modifications or high-pressure studies of the TTF–QBr_3_I single crystal would provide promising phase controls for magnetoelectric phenomena.

## Methods

### Crystallization of TTF CT complexes

Neutral CT complexes of 2:1 stoichiometry, (TTF)_2_(QBr_4–*n*_I_*n*_) (*n*=0–4), were obtained by slow evaporation or cooling (∼0 °C) of mixed acetonitrile solution of QBr_4–*n*_I_*n*_ and TTF as the main products. Occasionally, the 1:1 neutral complexes of TTF–QBr_2_I_2_ and TTF–QBrI_3_ crystallized as dark brown elongated plates, while the 1:1 ionic complexes of TTF–QBr_3_I and TTF–QBr_4_ crystallized as black rectangular plates. These minor crystal forms could be selectively crystallized with a size sufficient for electric measurements by prior seeding of several small single crystals. The 1:1 form of the TTF–QI_4_ complex was crystallized as elongated plates by slow evaporation or cooling of the toluene solution of the stoichiometric mixture of components.

### Electric, magnetic and optical measurements

Infrared absorption spectra of the samples dispersed in KBr disks (Supplementary Figs 2 and 3) were recorded at room temperature with a Fourier transform infrared spectrometer (JASCO FTIR-200) in a frequency range of 4,000−400 cm^−1^ at a resolution of 4 cm^−1^. The magnetization of the polycrystalline TTF–QBr_3_I powder wrapped in a thin aluminium foil was measured at a constant magnetic field of 0.2 T by a superconducting quantum interference device magnetometer (MPMS XL, Quantum Design). The spin susceptibility data were obtained with correction for the magnetization of a blank foil and the core diamagnetism using the compilation of Pascal's constant (2.2 × 10^−4^ e.m.u. mol^−1^). The temperature-dependent dielectric constant was measured with an LCR metre (HP 4284A) and at a cooling/heating rate of 1−2 K min^−1^. Hydrostatic pressure in the pressure-transmitting oil (Daphne 7,373, Idemitsu Kosan) was generated using a clamp-type high-pressure cell. The applied pressure at each transition point or *T*=5 K (Figs 2b and 3) was obtained by correcting the thermal change of the pressure (d*p*/d*T*=0.76 MPa K^−1^ for *T*>90 K and constant *p* for *T*<90 K) owing to the contraction of the medium^33^. The *P*–*E* hysteresis curves were measured at 1 kHz with a commercial ferroelectric tester (Precision Premier II, Radiant Technologies) equipped with a voltage amplifier (Precision 4 kV HVI, Radiant Technologies).

### Crystallographic studies

For the TTF–QBrI_3_ and (TTF)_2_(QBr_2_I_2_) crystals, both the X-ray diffraction data collection at room temperature and the assignment of the crystallographic axes of the bulk single crystals were completed using a four-circle diffractometer (Rigaku AFC7R; graphite-monochromated MoKα radiation) equipped with a charge-coupled device area detector. For the TTF–QBr_2_I_2_, TTF–QBrI_3_ and TTF–QI_4_ crystals, the X-ray diffraction experiments at room temperature were carried using a Rigaku DSC imaging plate diffractometer and synchrotron radiation (*λ*=0.6889 Å) at beamline BL-8A or BL-8B of the photon factory, High-Energy Accelerator Research Organization. The X-ray beam was monochromatized by a Si double-crystal monochromator and focused by a bent cylindrical mirror made of a Si crystal coated with Rh. The reflection intensity data were collected with the use of the Rapid-AUTO software package (Rigaku Corp.) and analysed with the Crystal Structure crystallographic software packages (Molecular Structure Corp. and Rigaku Corp.). The final refinements were done with anisotropic atomic displacement parameters for the non-hydrogen atoms and with calculated positions (with a fixed C–H distance of 0.95 Å) for the hydrogen atoms. Crystallographic data and experimental details of the CT complexes of TTF and QBr_4–*n*_I_*n*_ are summarized in Supplementary Table 2 and Supplementary Data 1–5.

The change in the lattice parameters under hydrostatic pressure was examined at room temperature for a TTF–QBr_2_I_2_ single crystal using synchrotron radiation and a clamp-type Be-cylinder high-pressure cell filled with Daphne 7373 oil as the pressure-transmitting medium^34^. The diffraction study of a TTF–QBr_2_I_2_ single crystal under hydrostatic pressure was performed using synchrotron radiation and a diamond anvil cell filled with a 4:1 methanol–ethanol mixture. Diffraction spots were recorded on the imaging plate using a diffractometer equipped with a closed-cycle He-gas refrigerator, and the diamond anvil cell was mounted on the cold head of the refrigerator. The pressure was evaluated from the lattice constant of a NaCl internal pressure marker.

## Additional information

**Accession codes:** The X-ray crystallographic coordinates for structures reported in this Article have been deposited at the Cambridge Crystallographic Data Centre (CCDC), under deposition number CCDC-1059244-1059248. These data can be obtained free of charge from the Cambridge Crystallographic Data Centre via www.ccdc.cam.ac.uk/data_request/cif.

**How to cite this article:** Horiuchi, S. *et al.* Quantum ferroelectricity in charge-transfer complex crystals. *Nat. Commun.* 6:7469 doi: 10.1038/ncomms8469 (2015).

## Supplementary Material

Supplementary Information Supplementary Figures 1-5, Supplementary Tables 1-2, Supplementary Discussion and Supplementary References

Supplementary Data 1 Crystallographic Data (TTF-QBr2I2 2:1)

Supplementary Data 2 Crystallographic Data (TTF-QBr2I2 1:1)

Supplementary Data 3 Crystallographic Data (TTF-QBr3I 1:1)

Supplementary Data 4 Crystallographic Data (TTF-QBrI3 1:1)

Supplementary Data 5 Crystallographic Data (TTF-QI4 1:1)

## References

[b1] Tokura Y. *et al.* Domain-wall dynamics in organic charge-transfer compounds with one-dimensional ferroelectricity. Phys. Rev. Lett. 63, 2405–2408 (1989). 10040880 10.1103/PhysRevLett.63.2405

[b2] Okamoto H. *et al.* Anomalous dielectric response in tetrathiafulvalene-*p*-chloranil as observed in temperature- and pressure-induced neutral-to-ionic phase transition. Phys. Rev. B Condens. Matter 43, 8224–8232 (1991). 9996449 10.1103/physrevb.43.8224

[b3] Girlando A., Painelli A., Bewick S. A. & Soos Z. G. Charge fluctuations and electron–phonon coupling in organic charge-transfer salts with neutral–ionic and Peierls transitions. Synth. Met. 141, 129–138 (2004).

[b4] Soos Z. G., Bewick S. A., Peri A. & Painelli A. Dielectric response of modified Hubbard models with neutral-ionic and Peierls transitions. J. Chem. Phys. 120, 6712–6720 (2004). 15267564 10.1063/1.1665824

[b5] Giovannetti G., Kumar S., Stroppa A., van den Brink J. & Picozzi S. Multiferroicity in TTF-CA organic molecular crystals predicted through *ab initio* calculations. Phys. Rev. Lett. 103, 266401 (2009). 20366325 10.1103/PhysRevLett.103.266401

[b6] Ishibashi S. & Terakura K. First-principles study of spontaneous polarization in tetrathiafulvalene-*p*-chloranil (TTF-CA). Phys. B Condens. Matter 405, S338–S340 (2010).

[b7] Horiuchi S., Hasegawa T. & Tokura Y. Molecular donor–acceptor compounds as prospective organic electronics materials. J. Phys. Soc. Jpn 75, 051016 (2006).

[b8] Horiuchi S. & Tokura Y. Organic ferroelectrics. Nat. Mater. 7, 357–366 (2008). 18432209 10.1038/nmat2137

[b9] Torrance J. B., Vazquez J. E., Mayerle J. J. & Lee V. Y. Discovery of a neutral-to-ionic phase transition in organic materials. Phys. Rev. Lett. 46, 253–257 (1981).

[b10] Aoki S., Nakayama T. & Miura A. Temperature-induced neutral-ionic transition in dimethyltetrathiafulvalene-*p*-chloranil. Phys. Rev. B Condens.. Matter 48, 626–629 (1993). 10006826 10.1103/physrevb.48.626

[b11] Kagawa F. *et al.* Electric-field control of solitons in a ferroelectric organic charge-transfer salt. Phys. Rev. Lett. 104, 227602 (2010). 20867204 10.1103/PhysRevLett.104.227602

[b12] Kobayashi K. *et al.* Electronic ferroelectricity in a molecular crystal with large polarization directing antiparallel to ionic displacement. Phys. Rev. Lett. 108, 237601 (2012). 23003988 10.1103/PhysRevLett.108.237601

[b13] Horiuchi S., Kobayashi K., Kumai R. & Ishibashi S. Ionic versus electronic ferroelectricity on donor-acceptor molecular sequence. Chem. Lett. 43, 26–35 (2014).

[b14] Monceau P., Nad F. Y. & Brazovskii S. Ferroelectric Mott-Hubbard phase of organic (TMTTF)_2_X conductors. Phys. Rev. Lett. 86, 4080–4083 (2001). 11328100 10.1103/PhysRevLett.86.4080

[b15] Girlando A., Pecile C. & Torrance J. B. A key to understanding ionic mixed stacked organic solids: tetrathiafulvalene-bromanil (TTF-BA). Solid State Commun. 54, 753–759 (1985).

[b16] Kagawa F., Horiuchi S., Tokunaga M., Fujioka J. & Tokura Y. Ferroelectricity in a one-dimensional organic quantum magnet. Nat. Phys. 6, 169–172 (2010).

[b17] Sachdev S. Quantum Phase Transitions Cambridge Univ. Press (2011).

[b18] Schneider T., Beck H. & Stoll E. Quantum effects in an *n*-component vector model for structural phase transition. Phys. Rev. B 13, 1123–1130 (1976).

[b19] Müller K. A. & Burkard H. SrTiO_3_: an intrinsic quantum paraelectric below 4K. Phys. Rev. B 19, 3593–3602 (1979).

[b20] Rytz D., Höchli U. T. & Bilz H. Dielectric susceptibility in quantum ferroelectrics. Phys. Rev. B 22, 359–364 (1980).

[b21] Itoh M. *et al.* Ferroelectricity induced by oxygen isotope exchange in strontium titanate perovskite. Phys. Rev. Lett. 82, 3540–3543 (1999).

[b22] Rowley S. E. *et al.* Ferroelectric quantum criticality. Nat. Phys. 10, 367–372 (2014).

[b23] Matsuzaki S., Hiejima T. & Sano M. Pressure-induced neutral–ionic phase transition of a tetrathiafulvalene–iodanil crystal. Bull. Chem. Soc. Jpn 64, 2052–2057 (1991).

[b24] Sadohara R. & Matsuzaki S. Neutral–ionic transition of charge transfer complexes of TTF and tetrahalo-*p*-benzoquinones. Mol. Cryst. Liq. Cryst. 296, 269–280 (1997).

[b25] Matsuzaki S., Hiejima T. & Sano M. Pressure-induced neutral-ionic transition in a 2:1 charge transfer crystal of tetrathiafulvalene and iodanil, (TTF)_2_IA. Solid State Commun. 82, 301–304 (1992).

[b26] Matsuzaki S. & Yartsev V. M. Charge distribution in mixed-valence trimer. Solid State Commun. 89, 941–944 (1994).

[b27] Mayerle J. J., Torrance J. B. & Crowley J. I. Mixed-stack complexes of tetrathiafulvalene. The structures of the charge-transfer complexes of TTF with chloranil and fluoranil. Acta Cryst. 35, 2988–2995 (1979).

[b28] Barrett J. H. Dielectric constant in perovskite type crystals. Phys. Rev. 86, 118–120 (1952).

[b29] Horiuchi S., Okimoto Y., Kumai R. & Tokura Y. Quantum phase transition in organic charge-transfer complexes. Science 299, 229–232 (2003). 12522245 10.1126/science.1076129

[b30] Okimoto Y., Horiuchi S., Saitoh E., Kumai R. & Tokura Y. Far-infrared optical response of neutral-ionic phase transition in an organic charge-transfer complex. Phys. Rev. Lett. 87, 187401 (2001).

[b31] Lemée-Cailleau M. H. *et al.* Thermodynamics of the neutral-to-ionic transition as condensation and crystallization of charge-transfer excitations. Phys. Rev. Lett. 79, 1690–1693 (1997).

[b32] Horiuchi S., Kumai R. & Tokura Y. Chemical control of ferroelectric neutral-ionic transition in charge-transfer complexes, TTF_1-*x*_TSF_*x*_QCl_4_ [TTF=tetrathiafulvalene; TSF=tetraselenafulvalene; QCl_4_=*p*-chloranil]. J. Am. Chem. Soc. 120, 7379–7380 (1998).

[b33] Murata K., Yoshino H., Yadav H. O., Honda Y. & Shirakawa N. Pt resistor thermometry and pressure calibration in a clamped pressure cell with the medium, Daphne 7373. Rev. Sci. Instrum. 68, 2490–2493 (1997).

[b34] Hasegawa T., Kumai R., Takahashi Y. & Tokura Y. Clamp-type pressure cell for full structure determination of molecular single crystals up to 1.5GPa. Rev. Sci. Instrum. 76, 073903 (2005).

