# Supplementary material for: Quantum ferroelectricity in charge-transfer complex crystals
Source: Nat Commun. 2015 Jun 16;6:7469. doi: 10.1038/ncomms8469 (PMC4490386; doi:10.1038/ncomms8469)
Supplement: Supplementary Information — Supplementary Figures 1-5, Supplementary Tables 1-2, Supplementary Discussion and Supplementary References [file ncomms8469-s1.pdf]

Supplementary Figures

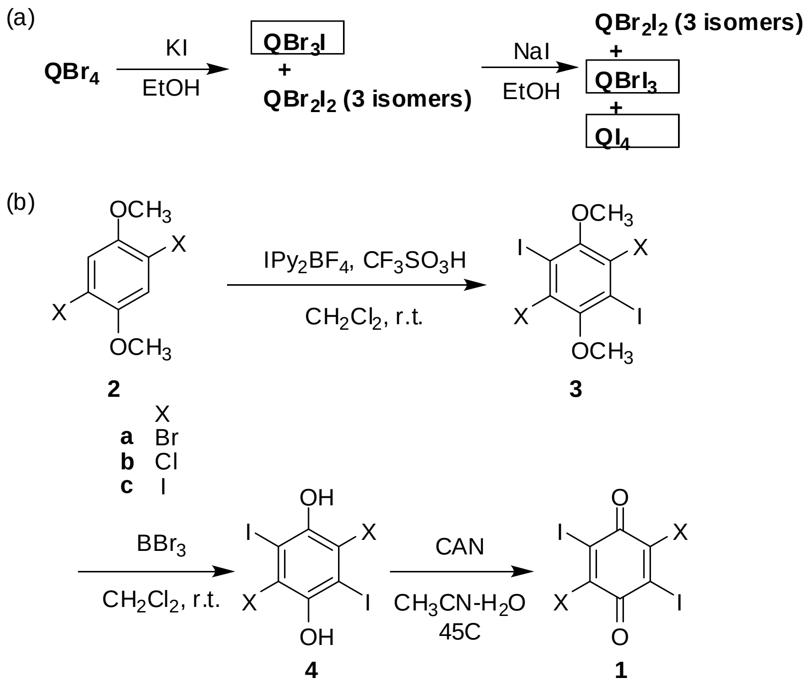

Supplementary Figure 1: Synthetic procedure of tetrahalo-*p*-benzoquinones ($QBr_{4-n}I_n$) with *n* iodine and 4–*n* bromine substituents. (**a**) Conventional synthetic procedure and isolation of $QBr_{4-n}I_n$ molecules by GPC from their mixture products. (**b**) Regioselective preparations of diiodo-substituted *p*-benzoquinones.

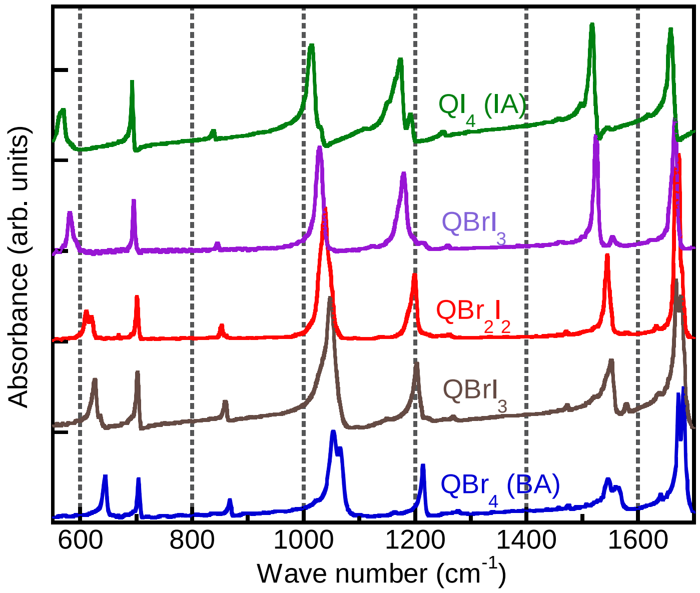

Supplementary Figure 2: Infrared absorption spectra of the powdered *p*-benzoquinones $QBr_{4-n}I_n$ dispersed in a KBr disk.

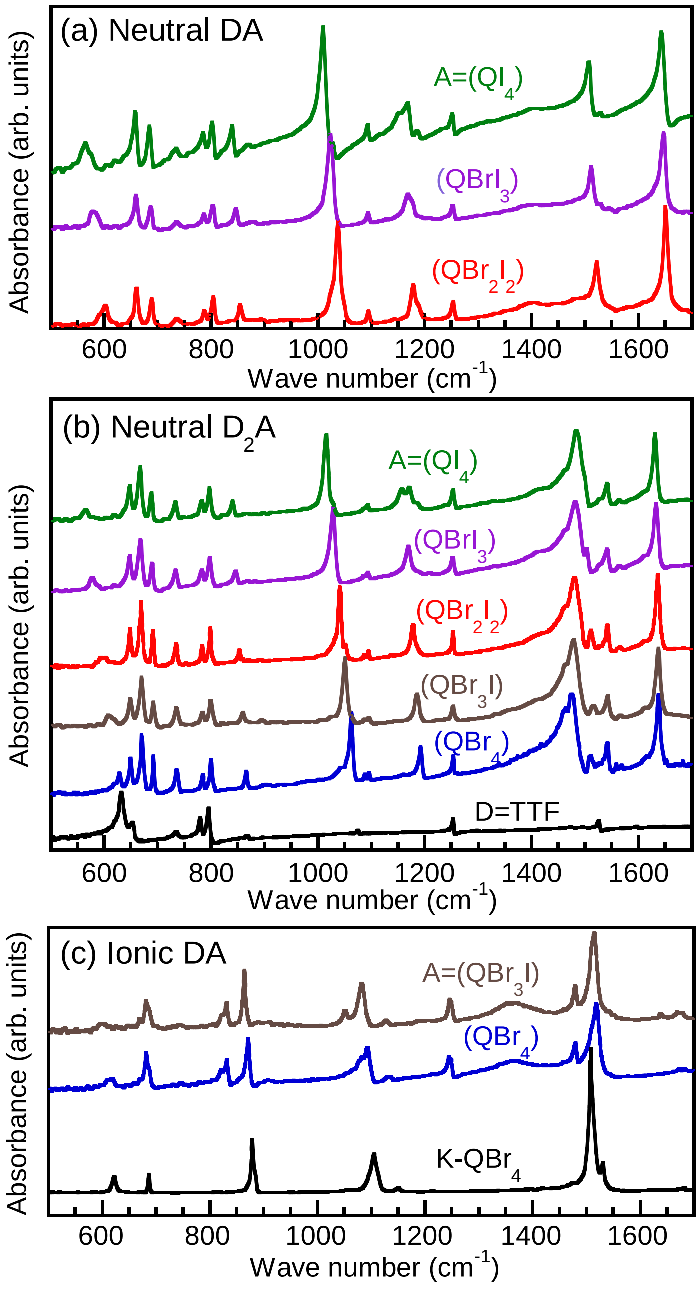

Supplementary Figure 3: Infrared absorption spectra of the powdered charge-transfer complexes of TTF and *p*-benzoquinones $QBr_{4-n}I_n$. (a) Neutral 1:1 complexes in comparison with neutral TTF. (b) Neutral 2:1 complexes. (c) Ionic 1:1 complexes in comparison with K-QBr_4 salt.

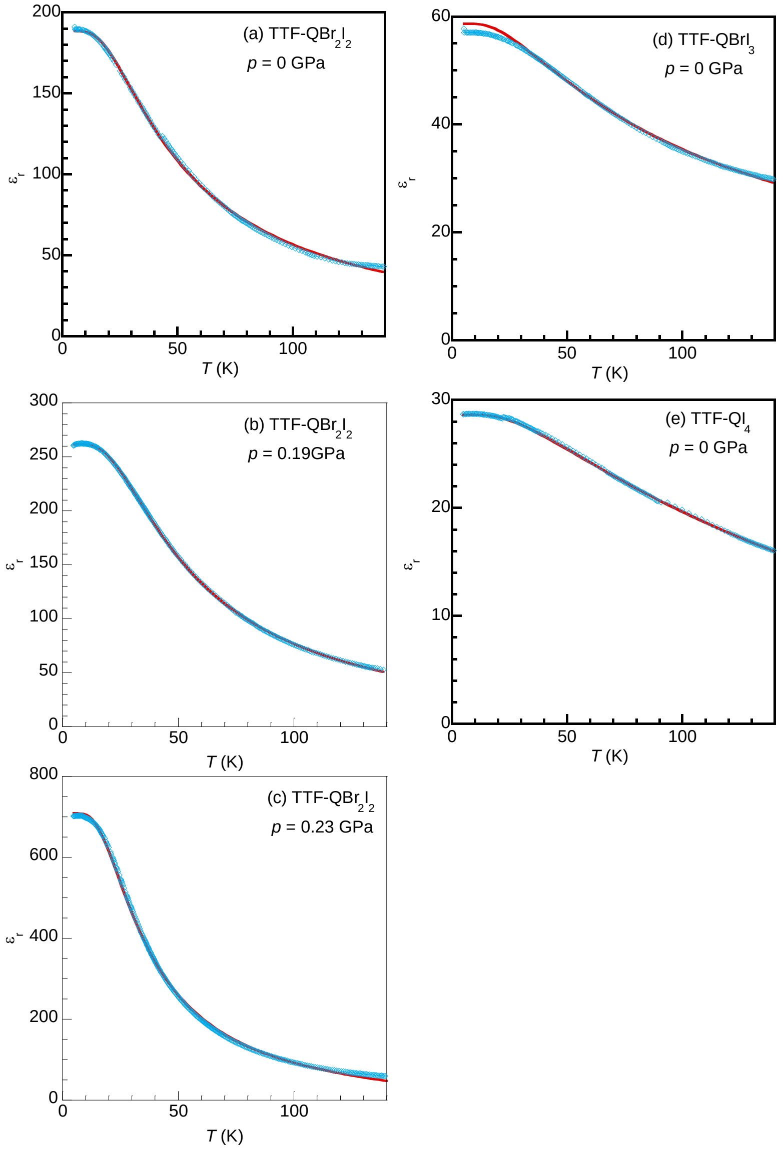

Supplementary Figure 4: Fittings of low-temperature permittivity data (blue dots) to the Barrett formula (solid red curve).

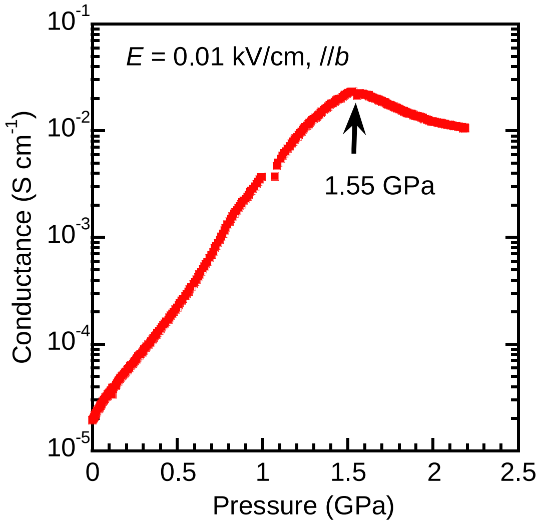

Supplementary Figure 5: Hydrostatic pressure dependence of the conductivity of a 1:1 TTF-QBr_2I_2 at room temperature (295 K). A dual structured clamp-type pressure cell was used in the conductivity measurements at room temperature. We used Daphne7474 oil as a pressure-transmitting medium.

Supplementary Tables

Supplementary Table 1: TTF and DMTTF complexes of tetrahalo-*p*-benzoquinones exhibiting NIT or SP. The complexes in the same box are isomorphous to each other in the crystal structure.

**Acceptor (A)**	TTF-**A** (1:1)	TTF-**A** (2:1)	DMTTF-**A** (1:1)
**QCl_4**	FE, TINIT (81 K) (monoclinic)	Non	AFE, TINIT (triclinic)
**$QBrCl_3$**	FE, TINIT (70 K) (monoclinic)	PINIT (monoclinic)	AFE, TINIT (triclinic)
**QBr_2Cl_2**	Non	PINIT (monoclinic)	AFE, NIT (QCP) (triclinic)
**QBr_3Cl**	Non	PINIT (monoclinic)	AFE, PINIT (triclinic)
**QBr_4**	FE, PM & SP (52 K) (triclinic)	PINIT (monoclinic)	AFE, PINIT (triclinic)
**QBr_3I**	PM (triclinic)	PINIT (monoclinic)	-
**QBr_2I_2**	FE, PINIT (near QCP) (triclinic)	PINIT (monoclinic)	-
**$QBrI_3$**	PINIT (triclinic)	PINIT (monoclinic)	-
**QI_4**	PINIT (triclinic)	PINIT (monoclinic)	-

FE = ferroelectric, AFE = antiferroelectric type, Non = no CT complex

NIT = neutral-to-ionic phase transition

TINIT = temperature-induced NIT (phase transition point at ambient pressure)

PINIT = pressure-induced NIT

SP = spin-Peierls -type phase transition, PM = paramagnetic

QCP = quantum critical point

Supplementary Table 2: Crystal data and experimental details of TTF-$QBr_{4-n}I_n$ crystals at room temperature.

	TTF-QBr_2I_2	TTF-$QBrI_3$	TTF-QI_4	TTF-QBr_3I	$TTF_2QBr_2I_2$
chemical formula	$C_{12}H_4Br_2I_2O_2S_4$	$C_{12}H_4Br_1I_3O_2S_4$	$C_{12}H_4I_4O_2S_4$	$C_{12}H_4Br_3I_1O_2S_4$	$C_{18}H_8Br_2I_2O_2S_8$
formula wt	722.02	769.02	816.02	675.02	926.36
Temperature /K	300	300	300	295	295
a/ Å	7.008(2)	7.0535(15)	7.0996(14)	8.4688(10)	10.5010(16)
b/ Å	7.571(2)	7.6622(17)	7.7287(14)	8.7721(12)	11.9699(15)
c/ Å	9.696(3)	9.7173(18)	9.7508(16)	11.6407(16)	11.0318(17)
α/deg	111.669(10)	111.757(13)	111.648(7)	93.405(4)	90
β/deg	108.287(7)	107.972(9)	107.843(7)	97.351(4)	109.896(5)
γ/deg	93.393(10)	94.140(13)	94.700(8)	91.892(5)	90
V/ $Å^3$	445.0(2)	453.50(16)	461.74(15)	855.4(2)	1303.9(4)
Crystal system	triclinic	triclinic	triclinic	triclinic	monoclinic
space group	P-1 (#2)	P-1 (#2)	P-1 (#2)	P-1 (#2)	$P2_1/n$ (#14)
ρ_{calc}/ g cm^{-3}	2.694	2.816	2.934	2.620	2.359
Z	1	1	1	2	2
dimensions / mm	0.20×0.15×0.15	0.20×0.15×0.10	0.20×0.03×0.03	0.38×0.20×0.10	0.22×0.17×0.11
radiation	Synchrotron (λ = 0.6889 Å)	Synchrotron (λ = 0.6889 Å)	Synchrotron (λ = 0.6889 Å)	MoKα	MoKα
$2\theta_{max}$/deg	79	144	144	55	55
R_{int}	0.031	0.020	0.030	0.032	0.028
reflcn used ($2\sigma(I)<I$)	2109	3357	5689	2686	2371
no. of variables	108	108	108	239	171
R	0.0365	0.0313	0.037	0.029	0.032
R_w	0.0398	0.0336	0.040	0.015	0.024
GOF	0.98	0.95	0.85	0.87	1.15

Supplementary Discussion

Preparation of Iodinated *p*-Benzoquinones: Outline.

The synthetic route is outlined in Supplementary Figure 1. The conventional synthesis of QI_4 has been a direct iodine substitution of QBr_4 with alkali iodide.[1,2] Its products contain incompletely substituted $QBr_{4-n}I_n$ molecules, which have not been satisfactorily separated from one another by the repetitive recrystallizations. The present investigation requires the $QBr_{4-n}I_n$ molecules in chemically pure form for systematic controls of dielectric and magnetic properties of their TTF complexes.

After the conventional iodine substitutions of QBr_4,[1,2] the $QBr_{4-n}I_n$ of different *n* were successfully separated from their mixture through the gel-permeation chromatography (GPC). This purification can work for isolating 2-iodo-3,5,6-tribromo-*p*-benzoquinone (QBr_3I) and 2-bromo-3,5,6-triiodo-*p*-benzoquinone ($QBrI_3$) as well as pure QI_4. On the other hand, the dibromodiiodo-*p*-benzoquinones, which cannot be separated into the three isomers, require a regioselective iodination procedure. Iodination of the commercially available 2,5-dibromo-1,4-dimethoxybenzene by bis(pyridine)iodonium(I)tetrafluoroborate (IPy_2BF_4) called the Barluenga's reagent[3] was carried out (35-36 % yield) according to the report on treating the dimethoxybenzene.[4] The subsequent demethylation with 2 equivalent boron tribromide at room temperature[5,6] gave the corresponding hydroquinone in quantitative yield (95-96 %). Oxidation of hydroquiones with cerium(IV) ammonium nitrate (CAN)[7] afforded the 2,5-dibromo-3,6-diiodo-*p*-benzoquinone (QBr_2I_2) (40 % yield). The same procedure starting from the 2,5-dichloro-1,4-dimethoxybenzene gave the 2,5-dichloro-3,6-diiodo-*p*-benzoquinone (QCl_2I_2) in the similar yield.

Synthesis

The commercially available 2,5-dihalo-1,4-dimethoxybenzenes and bis(pyridine)iodonium(I)tetrafluoroborate (IPy_2BF_4), BBr_3 solution, cerium(IV) ammonium nitrate (CAN) were of reagent grade. The purchased tetrathiafulvalene (TTF) was purified by repetitive recrystallizations and vacuum sublimation. Melting points were uncorrected. ^{1}H NMR spectra were recorded on a Bruker Avance NMR spectrometer at 500MHz. Chemical shifts (δ) are reported in ppm relative to tetramethylsilane (δ 0.00). Gas chromatographic/mass analyses (GC/MS) were performed on a Shimadzu GCMS-QP5000 in EI mode.

Iodination of *p*-Bromanil. Mixture of powdered QBr_4 (4.43 g, 10.5 mmol) and finely ground potassium iodide (1.76 g, 10.6 mmol) was refluxed in ethanol (500 mL) for 3 h, then cooled rapidly. The precipitate was filtrated and washed with water and then ethanol. At this stage, the brick red powder (3.43 g) contains QBr_3I and mixture of three isomers of QBr_2I_2, as well as unreacted QBr_4. The mixture of $QBrI_3$ and QI_4 has been obtained by twice treatments with iodide according to the literature method;[1] QBr_4 (41.8 g, 88.8 mmol) with KI (34 g, 205 mmol)

in ethanol (450 mL, refluxed 3 h), and then with NaI (29.1 g, 194 mmol) in ethanol (400 mL, refluxed 2 h) afforded 37.6 g of yellowish brown solid. After vacuum sublimation under temperature gradient or recrystallization from tolune, analytical samples of QBr_3I, $QBrI_3$, and QI_4 were obtained by gel-permeation chromatography (GPC) using toluene as eluent with JAIGEL 1H/2H column assembly (Japan Analytical Industry Co. ltd).

2-Iodo-3,5,6-tribromo-*p*-benzoquinone (QBr_3I). Red solid, mp 273.0-274.0 °C, *Anal.* Calcd for $C_6Br_3IO_2$: C, 15.31; O, 6.80. Found: C, 15.41; O, 7.02.

2-Bromo-3,5,6-triiodo-*p*-benzoquinone ($QBrI_3$). Brown solid, mp 263.4-263.9 °C, *Anal.* Calcd for $C_6BrI_3O_2$: C, 12.76; O, 5.67. Found: C, 12.84; O, 5.83.

***p*-Iodanil (QI_4)**. Dark brown solid, mp 297.2-297.8 °C, *Anal.* Calcd for $C_6I_4O_2$: C, 11.78; O, 5.23. Found: C, 11.83; O, 5.34.

Iodination of Dimethoxybenzenes. – *General procedure 1 (GP1).* Trifluoromethanesulfonic acid (4.2 eq) in dry dichloromethane was dropwise added to a stirred solution of 2,5-dihalo-1,4-dimethoxybenzene (**2**) and IPy_2BF_4 (2.1 eq) in dry dichloromethane over 10 minutes under argon atmosphere at room temperature. After stirring for 7 h, the reaction mixture was quenched with deionized water and extracted with dichloromethane. The organic phase was washed with aqueous $Na_2S_2O_3$, dried ($MgSO_4$), evaporated in vacuo to yield crude brownish powder of **3**. Recrystallization from ethanol and further purification by sublimation in vacuum afforded analytical samples.

2,5-Dibromo-3,6-diiodo-1,4-dimethoxybenzene (3a). GP1: **2a** (7.58 g, 25.6 mmol) and IPy_2BF_4 (20.46 g, 55.0 mmol) in CH_2Cl_2 (120 mL) with CF_3SO_3H (16.11 g, 107.3 mmol) in CH_2Cl_2 (30 mL). Colorless solid of **3a** (4.89 g, 35 %), mp 179.3-180.3 °C, ^{1}H NMR (500 MHz, $CDCl_3$): δ 3.83 (singlet, 6H), GC/MS (EI) m/z: 547.6, *Anal.* Calcd for $C_8H_6Br_2I_2O_2$: C, 17.54; H, 1.10. Found: C, 18.03; H, 1.03.

2,5-Dichloro-3,6-diiodo-1,4-dimethoxybenzene (3b). GP1: **2b** (2.84 g, 6.46 mmol) and IPy_2BF_4 (4.94 g, 13.3 mmol) in CH_2Cl_2 (45 mL) with CF_3SO_3H (4.03 g, 26.8 mmol) in CH_2Cl_2 (40 mL). Colorless solid of **3b** (2.27 g, 36 %), mp 145.0-145.2 °C, ^{1}H NMR (500 MHz, $CDCl_3$): δ 3.85 (singlet, 6H), GC/MS (EI) m/z: 457.8, *Anal.* Calcd for $C_8H_6Cl_2I_2O_2$: C, 20.94; H, 1.32. Found: C, 21.24; H, 1.24.

Tetraiodo-1,4-dimethoxybenzene (3c). GP1: **2c** (2.52 g, 13.7 mmol) and IPy_2BF_4 (10.61 g, 28.5 mmol) in CH_2Cl_2 (60 mL) with CF_3SO_3H (8.60 g, 57.3 mmol) in CH_2Cl_2 (20 mL). Colorless solid of **3c** (0.63g, 7%), mp 213.8-215.7 °C, ^{1}H NMR (500 MHz, $CDCl_3$): δ 3.80 (singlet, 6H), GC/MS (EI) m/z: 641.7, *Anal.* Calcd for $C_8H_6I_4O_2$: C, 14.97; H, 0.94. Found: C, 15.20; H, 0.88.

Demethylation. – *General procedure 2 (GP2).* To a stirred solution of BBr_3 (1.0mol/l; 3eq) in dichloromethane was dropwise for 20 min added purified

2,5-dihalo-3,6-diiodo-dimethoxybenzene in dry dichloromethane at room temperature. After 18 h of stirring, the reaction mixture was quenched with deionized water and extracted with diethyl ether three times. After drying ($MgSO_4$) and removal of the solvent, the colorless powder was dissolved in ethanol. Addition of deionized water precipitates colorless solids of **4**, which were used for the oxidation without further purifications.

2,5-Dibromo-3,6-diiodohydroquinone (4a). GP2: With **3a** (4.89 g, 8.93 mmol) in CH_2Cl_2 (105 mL), BBr_3 in CH_2Cl_2 (1.0 M, 27 mL). Colorless solids of **4a** (4.39 g, 95%). GC/MS (EI) m/z: 519.7.

2,5-Dichloro-3,6-diiodohydroquinone (4b). GP2: With **3b** (2.25 g, 4.90 mmol) in CH_2Cl_2 (60 mL), BBr_3 in CH_2Cl_2 (1.0 M, 14 mL). Colorless solids of **4b** (2.03 g, 96%). GC/MS (EI) m/z: 429.8.

Oxidation of Hydroquinones. – *General procedure 3 (GP3).* To a stirred solution of crude hydroquinone in acetonitrile was dropwise added aqueous solution of CAN (2.2 eq) for 1 min at 45 °C. After 1 h of stirring, to the suspension was extracted with dichloromethane and washed with water twice and dried ($MgSO_4$). After removal of the solvent, the crude product washed with ethanol remained as brick red powder for QBr_2I_2 or orange powder for QCl_2I_2. The analytically pure crystals were collected by vacuum sublimation in the temperature gradient.

2,5-Dibromo-3,6-diiodo-*p*-benzoquinone (QBr_2I_2) (1a). **4a** (4.33 g, 8.33 mmol) in acetonitrile (60 mL) with CAN (8.97 g, 16.4 mmol) in water (50 mL). Brick red solid of **1a** (1.73g, 40.1%), mp 272.0-273.2 °C, *Anal*. Calcd for $C_6Br_2I_2O_2$: C, 13.92; O, 6.18. Found: C, 14.29; O, 6.54.

2,5-Dichloro-3,6-diiodo-*p*-benzoquinone (QCl_2I_2) (1b). **4b** (0.96 g, 2.23 mmol) in acetonitrile (19 mL) with CAN (2.75 g, 5.0 mmol) in water (15 mL). Orange solid of **1b** (0.43g, 45.0 %), mp 273.3-273.6 °C, *Anal*. Calcd for $C_6Cl_2I_2O_2$: C, 16.81; O, 7.46. Found: C, 17.03; O, 7.85.

Complex Formation of TTF with tetrahalo-*p*-benzoquinones

The TTF complexes with the series of tetrahalo-*p*-benzoquinones exhibited several crystal forms of different stoichiometry and/or ionicity. In this work, neutral charge-transfer complexes of a 2:1 stoichiometry, $(TTF)_2(QBr_{4-n}I_n)$ (n = 0-4) were often obtained as the main products, while the minor ones are 1:1 neutral complexes of TTF-QBr_2I_2 and TTF-$QBrI_3$ and the 1:1 ionic complexes of TTF-QBr_3I and TTF-QBr_4. Many of these complexes have been found to undergo the temperature- or pressure-induced phase transition (NIT or SP). For the systematic view, the supplementary Table 1 summarized the brief features of structure and phase transition for the TTF and DMTTF complexes with the series of tetrahalo-*p*-benzoquinones $QBr_{4-n}I_n$ and QBr_nCl_{4-n}. As far as we know, there is at least an additional crystal form, i.e. the ionic polymorph of TTF-QCl_4, which is excluded from the table due to its uncharacterized crystal structure.

The $(TTF)_2(QCl_2I_2)$ complex was also obtained without polymorphs and is isomorphous in the infrared spectra to $(TTF)_2(QBr_{4-n}I_n)$ (not shown).

Infrared Spectra

The tetrahalo-*p*-benzoquinones of *n* iodine and 4-*n* bromine substituents (abbreviated as $QBr_{4-n}I_n$, n = 0-4) have been prepared in series in search of ferroelectric charge transfer (CT) complexes with tetrathiafulvalene (TTF). As shown in Supplementary Figure 2, the infrared absorption spectra of $QBr_{4-n}I_n$ are very similar from one another. The systematic red shift with increasing *n* observed in some mode frequencies indicates the corresponding vibrations involving the carbon-halogen bonds. Three different crystal forms of TTF complexes can be easily identified by the different infrared absorption spectra as depicted in Supplementary Figure 3.

The charge ($-\rho$) on $QBr_{4-n}I_n$ molecules in the TTF complexes was estimated by the frequency shift ($\Delta\omega$) of the infrared-active C=O stretch mode, which shows a very large ionization shift upon complete ionization ($\Delta\omega_{C=O}{}^{(1)} = -169$ and -164 cm^{-1} for QBr_4 and QI_4, respectively; potassium salts are used for the $\rho = 1$ standard).[8,9] Under assumption of the linear relationship between the frequency and ρ: $\Delta\omega_{C=O} = \rho\Delta\omega_{C=O}{}^{(1)}$, we got small or nearly full ionicity for the 1:1 TTF complexes; $\Delta\omega_{C=O}$ and ρ: TTF-QBr_2I_2, -19 cm^{-1}, 0.11; TTF-$QBrI_3$, -19 cm^{-1}, 0.11; TTF-QI_4, -16 cm^{-1}, 0.09; TTF-QBr_3I, -163 cm^{-1}, 0.98; TTF-QBr_4, -157 cm^{-1}, 0.93. Compared with 1:1 neutral complexes, the neutral 2:1 complexes $(TTF)_2A$ exhibit slightly more anionic character on $QBr_{4-n}I_n$; $\Delta\omega_{C=O}$ and ρ: A = QBr_4, -39 cm^{-1}, 0.23; QBr_3I, -41 cm^{-1}, 0.25; 25QBr_2I_2, -33 cm^{-1}, 0.20; $QBrI_3$, -32 cm^{-1}, 0.20; QI_4, -27 cm^{-1}, 0.16.

X-ray crystallographic data

Supplementary Table 2 summarizes the crystal data and experimental details for the TTF complexes of *p*-benzoquinones ($QBr_{4-n}I_n$). The partially iodinated quinone molecules (n = 1-3) exhibit orientational disorder so that each halogen atomic sites has fractional occupations of Br and I atoms. These occupation factors were refined under restraints on their sums. Each QBr_2I_2 molecule occupies an inversion center in the two kinds of neutral TTF complexes. The occupation of its iodine atoms between crystallographically independent two sites exhibits apparent preference (92/8) in the 1:1 complex in contrast to the almost equal distribution (45/55) in the 2:1 complex. Similarly, the neutral 1:1 TTF-$QBrI_3$ complex remains the relatively iodine-rich (I/Br = 91/8) and –poor sites (I/Br = 59/42). On the other hand, iodine atoms in the 1:1 ionic TTF-QBr_3I complex are nearly evenly occupied with their fraction ranging from 0.2 to 0.3 on all the halogen sites of two crystallographically independent molecules.

Supplementary References

1. Torrey, H. A. & Hunter, W. H. The action of iodides on bromanil. Iodanil and some of its derivatives. *J. Am. Chem. Soc.* **34**, 702-716 (1912).
2. Jackson, C. L. & Bolton, E. K. Octoiodoparaquinhydrone. *J. Am. Chem. Soc.* **36**, 301-308 (1914)
3. Barluenga, J., González, J. M., García-Martín, M. A. & Cambos, P. J. Polyiodination on benzene at room temperature a regioselective synthesis of derivatives. *Tetrahedron Lett.* **34**, 3893-3896 (1993).
4. López-Alvarado, P., Avendaňo, C. & Menéndez, J. C. Efficient, multigram-scale synthesis of three 2,5-dihalobenzoquinones. *Synth. Commun.* **32**, 3233-3239 (2002).
5. McOmie, J. W. F., Watts, M. L. & West, D. E. Demethylation of aryl methyl ethers by boron tribromide. *Tetrahedron* **24**, 2289-2292 (1968).
6. Hünig, S. *et al.* 2,5-Disubstituted N, N′-dicyanoquinone diimines (DCNQIs) − syntheses and redox properties. *Eur. J. Org. Chem.* **1998**, 335-348.
7. Essers, M. & Haufe, G. Chemical consequences of fluorine substitution. Part 4. Diels–Alder reactions of fluorinated *p*-benzoquinones with Dane's diene. Synthesis of fluorinated D-homosteroids. *J. Chem. Soc. Perkin Trans. 1* **2002**, 2719-2728.
8. Matsuzaki, S., Hiejima, T. & Sano, M. Pressure-induced neutral–ionic phase transition of a tetrathiafulvalene–iodanil crystal. *Bull. Chem. Soc. Jpn.* **64**, 2052-2057 (1991).
9. Girlando, A., Zanon, I., Bozio, R. & Pecile, C. Raman and infrared frequency shifts proceeding from ionization of perhalo-*p*-benzoquinones to radical anions. *J. Chem. Phys.* **68**, 22-31 (1978).
